# Hotspot relaxation time of NbN superconducting nanowire single-photon detectors on various substrates

**DOI:** 10.1038/s41598-018-20035-7

**Published:** 2018-01-24

**Authors:** Lu Zhang, Lixing You, Xiaoyan Yang, Junjie Wu, Chaolin Lv, Qi Guo, Weijun Zhang, Hao Li, Wei Peng, Zhen Wang, Xiaoming Xie

**Affiliations:** 1 State Key Laboratory of Functional Material for Informatics, Shanghai Institute of Microsystem and Information Technology, Chinese Academy of Sciences, Shanghai, 200050 China; 2 University of Chinese Academy of Sciences, Beijing, 100049 China; 3 Center for Excellence in Superconducting Electronics, Chinese Academy of Sciences, Shanghai, 200050 China

## Abstract

Hotspot relaxation time (*τ*_*th*_) is one of the essential parameter which defines the maximum count rate of superconducting nanowire single-photon detectors (SNSPDs). We studied the *τ*_*th*_ for NbN-based SNSPDs on various substrates using the two-photon detection method based on the pump-probe spectroscopy technique. We observed that *τ*_*th*_ strongly increased with increasing bias current in the two-photon detection regime. In addition, the minimum hotspot relaxation time (*τ*_*th*_)_min_ was not significantly affected by the bath temperature; this is different from the previous observations reported for WSi SNSPDs. In addition, a strong dependency of (*τ*_*th*_)_min_ on the substrate was found. The minimum (*τ*_*th*_)_min_ was 11.6 ps for SNSPDs made of 5.5-nm-thick NbN on MgO (100), whereas the maximum (*τ*_*th*_)_min_ was 34.5 ps for SNSPDs made of 7.5-nm-thick NbN on Si (100). We presented a direct correlation between the values of *τ*_*th*_ and degrees of disorder of NbN films grown on different substrates.

## Introduction

Superconducting nanowire single-photon detector (SNSPD) is one of the most promising detectors in the near-infrared region owing to its unique combination of high speed, high detection efficiency, small timing jitter, and low dark count rate^1,2^. These features make SNSPD attractive for a variety of applications, especially, quantum information processing^3,4^. In recent years, many experimental and theoretical studies have investigated the detection mechanism to further enhance the count rates^5–7^. The difficulty in achieving high-count-rate SNSPDs stems from their unique internal mechanism of photon detection and the external readout circuit.

A typical SNSPD comprises a meandered nanowire patterned from few-nanometer-thin superconducting films such as NbN or WSi^8,9^. Absorption of a photon in a superconducting nanowire resulted in a non-equilibrium distribution of quasiparticles, with highly energetic hot electrons at a higher temperature than the Cooper pairs. The energy of the excited quasiparticles is redistributed within the electron and phonon system, which lead to an accumulation of quasiparticle excitations just above the energy gap and bring on the formation of a growing hotspot. The phonons in the nanowire dissipate the excess energy into the substrate and relax to the bath temperature; the equilibrium is restored throughout the entire superconducting sample then the hotspot disappears. The process of energy redistributed after the initial absorption of a photon is very fast (~fs) in disordered superconductors. Therefore, the energy transfer and down-conversion process following the absorption of a photon related to the formation and evolution of hotspot (hotspot relaxation process) can be characterized by a single time constant *τ*_*th*_ (hotspot relaxation time). The minimum time of SNSPD required for recovery to superconducting state is determined by the hotspot relaxation process. NbN-based SNSPDs have demonstrated intrinsic response times down to a few tens of ps, allowing the count rates to approach the GHz regime^10^. However, being limited by their large kinetic inductance (*L*_*k*_) and the input impedance of the readout circuit (*R*_*s*_), the devices have reset times that are several orders of magnitudes larger (~ns and larger) than their intrinsic response time. Reducing *L*_*k*_ and/or increasing *R*_*s*_ lead to a high count rate; however, the improvement in count rate is further restricted by the negative electro-thermal feedback^11^. In addition, for superconducting materials with slow hotspot relaxation process, devices would latch into a finite voltage state instead of self-resetting to the superconducting state after detecting a photon^12^. Therefore, *τ*_*th*_ is an important parameter that defines the maximum achievable count rate.

To quantitatively study hotspot relaxation process, several experiments have been conducted. In 2012, Heeres *et al*. proposed a quantum pump-probe technique based on the use of correlated photons from spontaneous parametric down-conversion (SPDC); they derived that the *τ*_*th*_ of an approximately 4-nm-thick NbN film grown on a sapphire substrate is ~15 ps and independent of the SPDC pump power and bias current^13^. In 2013, Zhou *et al*. demonstrated a novel method to measure ultrasensitive N-photon interferometric autocorrelation and determined that the *τ*_*th*_ of an approximately 4.3-nm-thick NbN film prepared on GaAs substrates is ~20 ps^14^. In 2016, Marsili *et al*. proposed a new technique that combined the pump-probe detection technology and Mach-Zehnder interferometer. They found that the *τ*_*th*_ of ~5-nm-thick WSi film on SiO_2_/Si substrates varied from ~80 ps to ~800 ps with increasing bias current (*I*_*b*_), bath temperature (*T*_*B*_), or photon energy^15^.

To identify the key factors affecting the relaxation time of NbN SNSPDs, we systematically studied the *τ*_*th*_ of NbN SNSPDs via two-photon detection experiments based on the pump-probe spectroscopy technique. Devices with various geometric parameters (e.g., film thickness and nanowire linewidth) were fabricated on different substrates: MgO (100), MgF_2_ (110), Al_2_O_3_ (0001), Si (100), and SiO_2_/Si (100). The *τ*_*th*_ values of different SNSPDs were measured and compared. Besides, the temperature dependence (2.15–5 K) of *τ*_*th*_ was also analyzed.

## Results

### Determination of *τ*_*th*_

SNSPD devices with different nanowire linewidth/space ratio (*w*/*s*) were fabricated from NbN films deposited on different substrates and cooled down for the characterization (see Table 1). Detailed information concerning device preparation, characterization and measurement setup can be found in the methods’ section below. The single-photon regime in SNSPD can be achieved when *I*_*b*_ is biased close to the switching current (*I*_*sw*_). When lowering *I*_*b*_, the energy of a single photon is insufficient to create a response pulse and the detector only responds to two or more photons^10,14,15^. Herein, we introduced the two-photon detection method, wherein the response pulse is triggered when the two incident photons separated by the time delay (*t*_*D*_) generate two hotspots that overlap spatially and temporally. According to recent research, the probability of having two absorbed photons of incident photons can be given by (*ημ*)^2^/2, where *η* is the two-photon detection efficiency and *μ* is the average number of photons per pulse^15,16^. When *t*_*D*_ was longer than *τ*_*th*_, the response probability is approximately equal to the sum of each pulse of (*ημ*)^2^. When *t*_*D*_ was shorter than *τ*_*th*_, the increased mean photon number doubled the response probability to 2 (*ημ*)^2^. Consequently, the evolution of hotspot relaxation process differed from that of SNSPD recovery.

**Table 1:** Substrate material, film thickness (*d*), nanowire linewidth (*w*), space (*s*), switching current (*I*_*sw*_), switching current density (*J*_*sw*_), and minimum hotspot relaxation time (*τ*_*th*_)_min_ for the seven SNSPDs.

Sample	Substrate material	*d* (nm)	*w* (nm)	*s* (nm)	*I*_*sw*_ (μA)	*J*_*sw*_ (MA/cm^2^)	(*τ*_*th*_)_min_ (ps)
#1	MgO (100)	5.5	90	110	33	6.7	11.6 ± 0.1
#2	MgO (100)	5.5	105	110	40	6.9	12.2 ± 0.1
#3	MgO (100)	5.5	120	110	51.5	7.8	12.6 ± 0.1
#4	MgF_2_ (110)	4.5	90	110	22	5.4	19.7 ± 0.1
#5	Al_2_O_3_ (0001)	7	130	70	21.5	2.4	22.5 ± 0.7
#6	SiO_2_/Si (100)	7	90	110	21	3.3	22.7 ± 0.1
#7	Si (100)	7.5	90	110	19	2.8	34.5 ± 0.3

**Figure 1:**
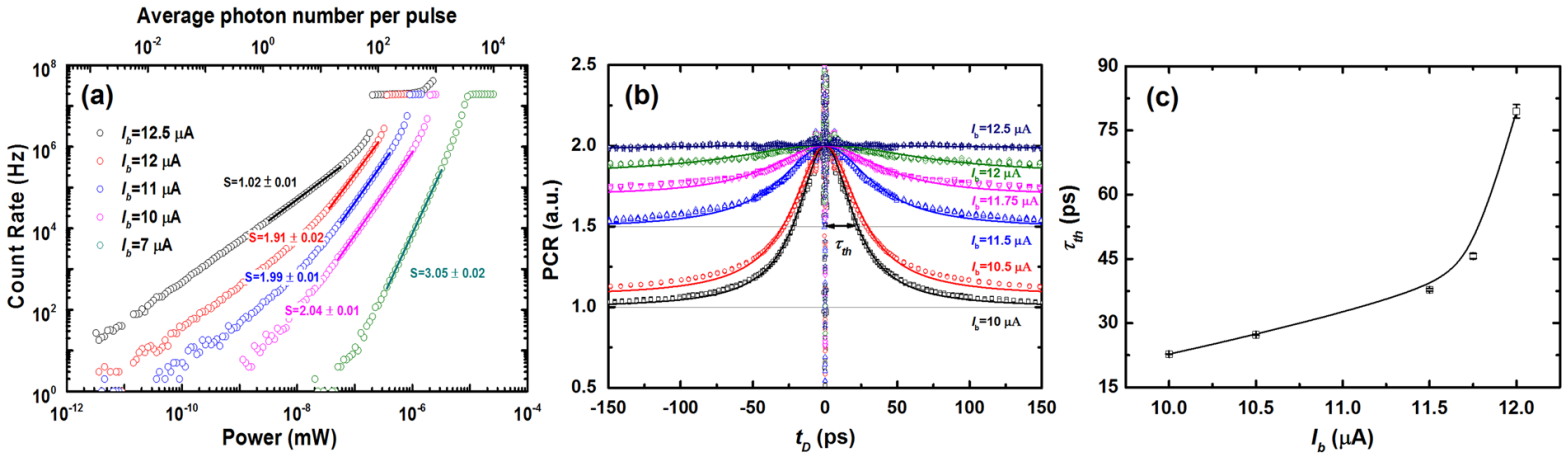
(**a**) Count rates as a function of light power (average photon number per pulse) at five different *I*_*b*_ values. The measured points, denoted by the open circles, were fitted with straight lines in the log–log scale. The slopes of the fitting lines are 1.02 ± 0.01, 1.91 ± 0.02, 1.99 ± 0.01, 2.04 ± 0.01, and 3.05 ± 0.02, respectively. (**b**) Normalized PCR as a function of *t*_*D*_, which is measured when the detector operated in the two-photon detection regime at *I*_*b*_ values of 10, 10.5, 11.5, 11.75, and 12 μA; *I*_*b*_ of 12.5 μA corresponding to the one-photon detection regime is also shown for comparison. The measured data were fitted with a Lorentz function. The black arrow denotes the half width at half maximum of the curve, which directly corresponds to *τ*_*th*_ measured at *I*_*b*_ = 10 μA. (**c**) *τ*_*th*_ as a function of *I*_*b*_ extracted from the data in (**b**).

First, Sample #6 was tested (7-nm-thick NbN/SiO_2_/Si (100) and 90-nm-wide nanowire with a 110-nm space) at *T*_*B*_ of 2.15 K. The *I*_*sw*_ of the device was approximately 21 μA. To distinguish the bias current range for two-photon detection, the count rates were measured as a function of the light power (average photon number per pulse) at several *I*_*b*_ values of 12.5 μA, 12 μA, 11 μA, 10 μA, and 7 μA. As shown in Fig. 1(a), the solid lines are fits to the measured data in the log-log scale. At *I*_*b*_ = 12.5 μA, and 7 μA, the fitted slope of 1.02 ± 0.01, and 3.05 ± 0.02 indicating that the detector operates in the single, and three-photon detection regime. At *I*_*b*_ of 12 μA, 11 μA, and 10 μA, the slopes of the fitting lines are 1.91 ± 0.02, 1.99 ± 0.01, and 2.04 ± 0.01, respectively. Therefore, the detector is dominated by two-photon detection for bias current 10 μA ≤ *I*_*b*_ ≤ 12 μA. We note that, there is no sharp boundary between either single or two-photon detection regime (~12 μA), and two-photon and three-photon regime (~10 μA). However, with the current lower than 10 μA, the count rate is too low to study with the fixed average photon number. With the current higher than 12.0 μA, the single-photon detection will be dominant so we cannot obtain the accurate*τ*_*th*_ based on Lorentz function fitting (see Fig. 1(b)). Therefore, the bias range of 10–12 μA is suitable for studying the *τ*_*th*_. Figure 1(b) illustrates the photon-response count rate (PCR) as a function of *t*_*D*_ at different *I*_*b*_ values from 10 μA to 12.5 μA. The measured data were fitted with a Lorentz function and normalized to 2 at *t*_*D*_ = 0 ps, ignoring the optical interference in the range −2 ps ≤ *t*_*D*_ ≤ 2 ps. At *I*_*b*_ = 12.5 μA, the PCR was irrelevant to *t*_*D*_ as the detector was nearly working in the one-photon detection regime. When *I*_*b*_ was lowered to 10 μA, the normalized PCR declined from 2 to ~1, with *t*_*D*_ increasing from 0 ps to ~150 ps^15,17^. In particular, the half width of this peak directly corresponds to *τ*_*th*_. Figure 1(c) shows *τ*_*th*_ as a function of *I*_*b*_ extracted from the data in Fig. 1(b). The minimum value of the hotspot relaxation time (*τ*_*th*_)_min_ was 22.7 ps ± 0.1 ps at *I*_*b*_ = 10 μA. Additionally, *τ*_*th*_ increased to 79.5 ps ± 1.5 ps, whereas *I*_*b*_ increased to 12 μA. These trends have also been reported for WSi SNSPDs and NbN waveguide-integrated SNSPDs, for which self-recombination rather than diffusive expansion explained the strong dependence of *τ*_*th*_ on *I*_*b*_^18^.

### Dependence of *τ*_*th*_ on bath temperature

**Figure 2:**
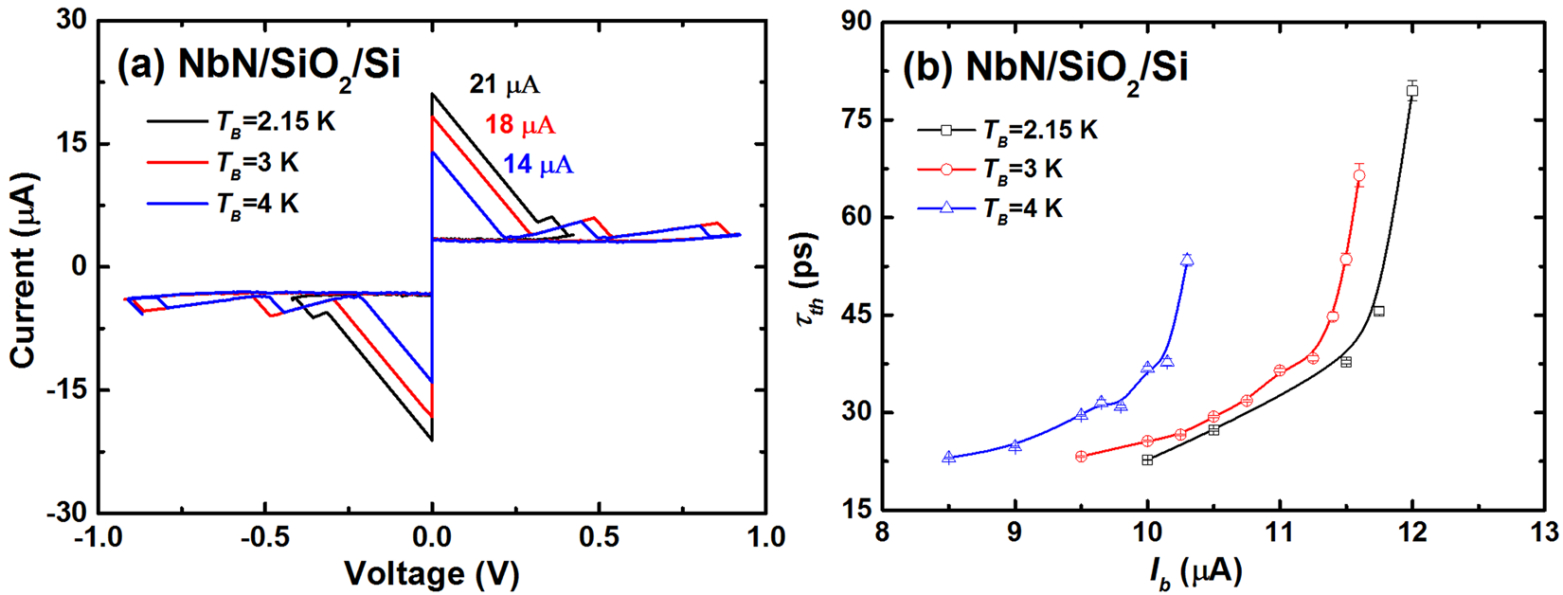
(**a**) *I-V* curves and (**b**) *τ*_*th*_ as a function of *I*_*b*_ measured at *T*_*B*_ values of 2.15 K, 3 K, and 4 K, respectively, for Sample #6.

To investigate the temperature dependence of *τ*_*th*_, we tuned the *T*_*B*_ of SNSPDs from 2.15 K to 4 K using the resistive heater. Figure 2(a) shows the current-voltage (*I-V*) characteristics of Sample #6 at three different temperatures (2.15 K, 3 K, and 4 K). *I*_*sw*_ decreased from 21 μA to 14 μA with increasing *T*_*B*_. As a result, the two-photon detection ranges changed to 10 μA ≤ *I*_*b*_ ≤ 12 μA (2.15 K), 9.5 μA ≤ *I*_*b*_ ≤ 11.6 μA (3 K), and 8.5 μA ≤ *I*_*b*_ ≤ 10.3 μA (4 K). Then, the *I*_*b*_ dependence of *τ*_*th*_ at different *T*_*B*_ values were studied; *τ*_*th*_ gradually increased with *I*_*b*_ and presented a considerable change around the one-photon detection regime for all *T*_*B*_ values (Fig. 2(b)). However, the *τ*_*th*_-*I*_*b*_ curve shifted to a smaller *I*_*b*_ with increasing *T*_*B*_ owing to the change of the two-photon detection range. In addition, at a fixed *I*_*b*_, *τ*_*th*_ increased with *T*_*B*_; this could be attributed to the decreased electron relaxation rate at high *T*_*B*_. However, the (*τ*_*th*_)_min_ values at *T*_*B*_ values of 2.15 K, 3 K, and 4 K were measured to be 22.7 ps ± 0.1 ps, 23.2 ps ± 0.1 ps, and 23.0 ps ± 0.1 ps, respectively. The results agree well with each other, and we saw a saturated trend of *τ*_*th*_ with decreasing the current, suggesting that the minimum hotspot relaxation time might be independent of *T*_*B*_. However, it is difficult to verify it by further decreasing the current since the device no longer worked in the two-photon detection regime anymore. This is different from the results reported for WSi SNSPDs, where (*τ*_*th*_)_min_ increased with *T*_*B*_ from 250 mK to 2 K^15^. Possibly, the smaller *I*_*sw*_ of WSi SNSPDs at higher temperatures restricts the ability to reach the lowest bias in the two-photon detection regime owing to the limit of the readout circuit. In other words, the (*τ*_*th*_)_min_ of WSi SNSPDs might be smaller at high temperatures as well.

### Dependence of *τ*_*th*_ on nanowire linewidth

It has been well established that the *w* of nanowire affects not only the optical absorption but also the intrinsic detection efficiency of SNSPD^8,19^. To understand the influence of *w* on *τ*_*th*_, three different SNSPDs were fabricated on MgO substrate, wherein *w* was designed to be 90, 105, and 120 nm, respectively. The *s* value was set to 110 nm. The measured *w*/*s* is consistent with the design as the EBL and RIE processes were optimized. The other parameters are listed as Samples #1–3 in Table 1.

**Figure 3:**
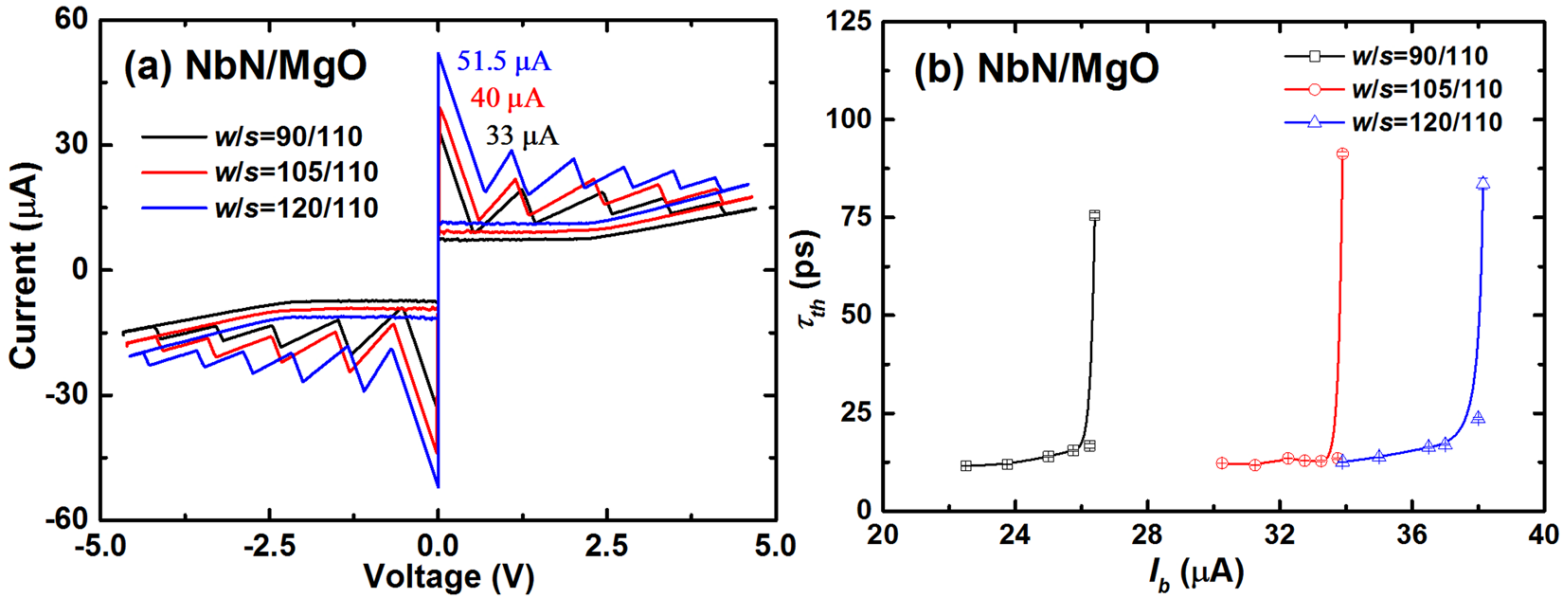
(**a**) *I-V* curves and (**b**) *τ*_*th*_ as a function of *I*_*b*_ at *T*_*B*_ values of 2.15 K for Sample #1–3 with different *w/s*.

Figure 3(a) shows the *I-V* curves of the SNSPDs with three different *w* values (90, 105, and 120 nm) that give *I*_*sw*_ values of 30, 40, and 51.5 μA respectively. Similarly, we obtained the relation of *τ*_*th*_ as a function of *I*_*b*_ for the three SNSPDs, as shown in Fig. 3(b). The trend displayed in the figure is comparable to that of Sample #6, and the curve shifts to the right with increasing *w* while maintaining the shape similarity. The (*τ*_*th*_)_min_ values for the three SNSPDs were determined to be 11.6 ps ± 0.1 ps, 12.2 ps ± 0.1 ps, and 12.6 ± 0.1 ps, respectively. The deviations were lesser than 1 ps. The slight increase in (*τ*_*th*_)_min_ with increasing *w* is explained by the presence of less thermal relaxation channels in the center of the nanowire as opposed to at the sides of the nanowire. In addition, the values are only half of those obtained for Sample #6; this is further discussed in the next section.

### Dependence of *τ*_*th*_ on the substrate

A variety of substrates, such as Si, MgO, MgF_2_, and Al_2_O_3_, have been used to grow ultrathin NbN films for fabricating SNSPDs^20–22^. To investigate the effect of substrates on *τ*_*th*_, we measured and compared the *τ*_*th*_ of NbN SPSPDs on different substrates [Si (100), MgO (100), MgF_2_ (110), Al_2_O_3_ (0001), and SiO_2_/Si (100)].

**Figure 4:**
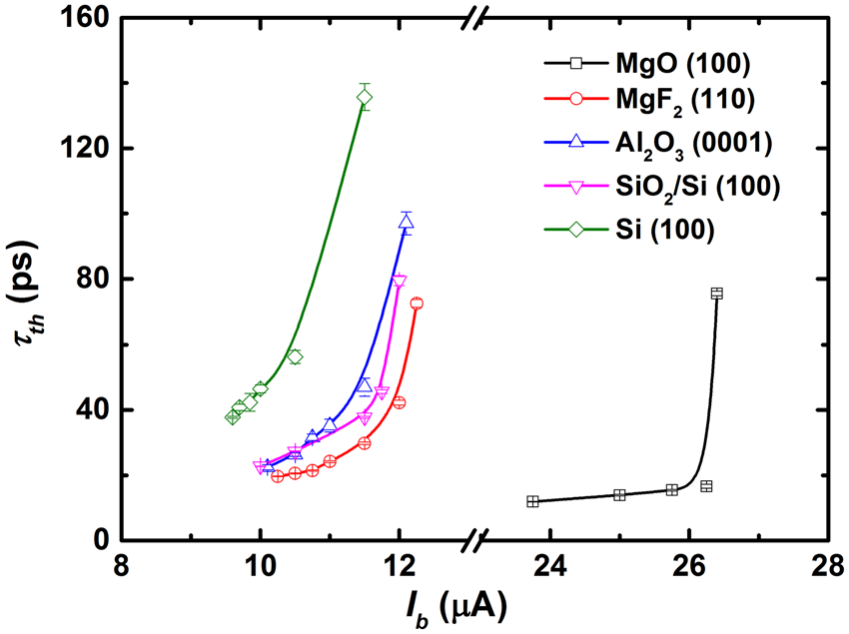
*τ*_*th*_ as a function of *I*_*b*_ at *T*_*B*_ values of 2.15 K for various substrates.

Figure 4 shows *τ*_*th*_ as a function of *I*_*b*_ at *T*_*B*_ = 2.15 K for SNSPDs on various substrates. All SNSPDs showed a similar increase in *τ*_*th*_ with increasing *I*_*b*_. The (*τ*_*th*_)_min_ values are summarized in Table 1 and follow the simple relation (*τ*_*th*_)_min_(MgO) ≤ (*τ*_*th*_)_min_(MgF_2_) ≤ (*τ*_*th*_)_min_(Al_2_O_3_) ≤ (*τ*_*th*_)_min_(SiO_2_/Si) ≤ (*τ*_*th*_)_min_(Si). The maximum difference of (*τ*_*th*_)_min_ is roughly three times. The smallest (*τ*_*th*_)_min_ (11.6 ps ± 0.1 ps) was obtained from the MgO (100) substrate, and the largest (*τ*_*th*_)_min_ (34.5 ps ± 0.3 ps) was obtained from the Si (100) substrate. Typically, *τ*_*th*_ is controlled by carrier diffusion as well as by the recombination time of the quasiparticles, which is indeed related to the degrees of disorder of the materials^15,23^. In other words, a film with a lower disorder would have a smaller (*τ*_*th*_)_min_.

### Role of disorder on *τ*_*th*_

**Figure 5:**
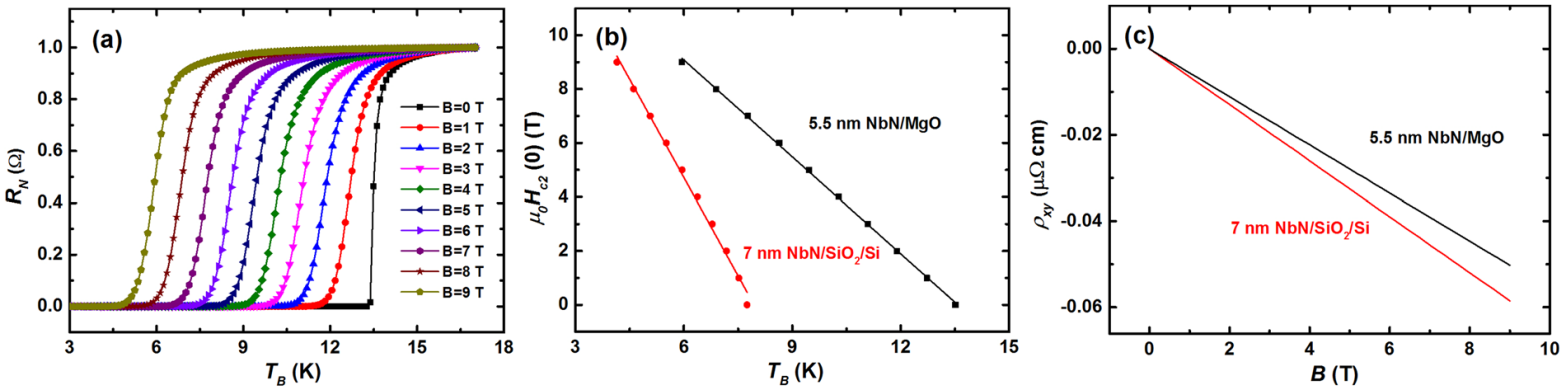
(**a**) *R*_*N*_ as a function of *T*_*B*_ for 5.5-nm-thick NbN on MgO (100) under different magnetic fields. (**b**) *μ*_0_*H*_*c2*_ as a function of *T*_*B*_ for 5.5-nm-thick NbN on MgO (100) and 7-nm-thick NbN on SiO_2_/Si (100). The solid lines are linear fits to the data. (**c**) *ρ*_*xy*_ as a function of *B* at 25 K for the two samples.

The superconducting properties of NbN films are determined by many parameters of the films. One of the key parameters is actually the film crystal structure, which is strongly influenced by not only the substrate material but many other parameters including the deposition condition. Meanwhile, NbN is a conventional s-wave superconductor and the superconducting properties of the NbN ultrathin film can be partially characterized by its degrees of disorder, which is related to not only the substrate material, but also the film thickness. The degrees of disorder of NbN films on two substrates MgO and SiO_2_/Si were quantitatively analyzed. First, we measured the normalized resistance (*R*_*N*_) as a function of *T*_*B*_ for the 5.5-nm-thick NbN/MgO and 7-nm-thick NbN/SiO_2_/Si substrates under different magnetic fields via transport measurements using a physical property measurement system. To accurately determine the resistivity (*ρ*_*n*_), the electrical resistance (*R*) was measured on a 4 μm × 40 μm microbridge using the standard four-probe method. Figure 5(a) shows the *R*_*N*_*-T*_*B*_ relations for the 5.5-nm-thick NbN/MgO sample. The upper critical field (*H*_*c2*_) values at different temperatures were then determined at the point where *R*_*N*_ becomes 50% of its normal value. The *μ*_0_*H*_*c2*_*-T*_*B*_ relations for the two samples are shown in Fig. 5(b). In the dirty limit of a superconductor film e.g., NbN, the mean electronic free path (*l*) is shorter than the Ginzburg–Landau coherence length (*ξ*_*GL*_). Then, *H*_*c2*_ (0), *ξ*_*GL*_, and the electron diffusion coefficient (*D*) are obtained from ${H}_{{c}2}({0})={0.69}{T}_{c}(d{H}_{{\rm{c2}}}/{dT}){|}_{{T}={T}_{c}},{\xi }_{{GL}}=\sqrt{{{\rm{\Phi }}}_{0}/{2}\pi {H}_{{c}{2}}({0})}$, and ${D}=-{4}{k}_{B}/({\pi }{ed}{H}_{{c}2}/{dT})$, respectively, where Φ_0_ is the flux quantum, *k*_*B*_ is the Boltzmann constant, and *e* is the electron charge^24,25^. For NbN films epitaxially grown on the MgO substrate, the obtained *D* value was 0.92 cm^2^/s, which is approximately twice the value of the film on the SiO_2_/Si substrate (0.47 cm^2^/s). The larger value of *D* suggests the presence of fewer defects and vacancies in the samples. The calculated *ξ*_*GL*_ values were 5.43 nm and 5.12 nm for the 5.5-nm-thick NbN/MgO and 7-nm-thick NbN/SiO_2_/Si samples, respectively.

Next, Hall measurement was performed using a four-probe AC technique. Figure 5(c) shows the Hall resistivity (*ρ*_*xy*_) as a function of *B* for the two samples measured at 25 K. The electron density (*n*) and the Ioffe–Regel parameter (*k*_*F*_*l*) are calculated from ${n}=1/{R}_{H}e,\,{k}_{F}={(3{\pi }^{2}n)}^{1/3}$, and $l={\rho }_{n}n{e}^{2}/\hslash {k}_{F}$, respectively, where ${R}_{H}={\rho }_{{xy}}/H$ is determined from the slope shown in Fig. 5(c). All the obtained values are listed in Table 2. As expected, $l < {\xi }_{{GL}}$, indicating that all NbN films are in the dirty limit irrespective of which substrate is used. However, the *k*_*F*_*l* values described herein range from moderately disordered (5.31) to strongly disordered (1.53), suggesting that NbN grown on the MgO (100) substrates exhibits a less disordered structure. The film superconductivity decreases with increasing disorder. The *J*_*sw*_ values listed in Table 1 are also confirmed the above judgments to some extent.

**Table 2:** Critical temperature (*T*_*c*_), normal resistivity (*ρ*_*n*_), upper critical field [*H*_*c2*_(0)], Ginzburg–Landau coherence length (*ξ*_*GL*_), electron diffusion coefficient (*D*), mean electronic free path (*l*), and Ioffe–Regel parameter (*k*_*F*_*l*) for the 5.5-nm-thick NbN/MgO (100) and 7-nm-thick NbN/SiO_2_/Si (100) samples.

Substrate material	*d* (nm)	*T*_*c*_ (K)	*ρ*_*n*_ (μΩ cm)	*μ*_0_*H*_*c2*_ *(0)* (T)	*ξ*_*GL*_ (nm)	*D* (cm^2^/s)	*l* (Å)	*k*_*F*_ *l*
MgO (100)	5.5	13.51	154	11.17	5.43	0.92	3.56	5.31
SiO_2_/Si (100)	7	7.71	562	12.56	5.12	0.47	1.08	1.53

## Discussion

The minimum (*τ*_*th*_)_min_ measured in our NbN SNSPDs is about only one in seven of WSi SNSPDs’, indicating a significant difference in material properties. The direct method to decrease *τ*_*th*_ is to decrease the degrees of disorder of the materials. However, how to decrease *τ*_*th*_ together with optimizing other parameters such as intrinsic detection efficiency still remains an open question, which is very interesting for not only understanding the mechanism of SNSPD but also the practical SNSPD with high performance.

In summary, the *τ*_*th*_ of NbN SNSPDs on various substrates were investigated with a two-photon detection measurement technique. The experimental setup was based on pump-probe spectroscopy, wherein the laser pulses were separated by time delays tuned in a wide range. To realize two-photon detection in a reasonable bias range, the typically average photon number per pulse is set as 40. The high average photon number per pulse guarantee the data collection in a reasonable time (one dot per hour) compared with the correlated photon-pair source based on SPDC.

Our results show that the bath temperature and nanowire linewidth do not significantly affects (*τ*_*th*_)_min_, i.e. they contribute less to the intrinsic upper limit of the count rate of SNSPD. However, (*τ*_*th*_)_min_ is strongly affected by the substrate because the degrees of disorder of NbN films are different. Epitaxially grown 5.5-nm-thick NbN on MgO (100) has a minimum (*τ*_*th*_)_min_ of 11.6 ps, and amorphous 7.5-nm-thick NbN on Si (100) has a maximum (*τ*_*th*_)_min_ of 34.5 ps. As a result, it is important to choose a suitable substrate if one wants to find lower *τ*_*th*_. Besides, one may further study NbN films deposited on different substrates with different deposition conditions to further identify the minimum of *τ*_*th*_. It may guide the further studies in the community on not only the physics of ultrathin superconducting films, but also the practical SNSPD with high count rate. Beyond that, the results are also interesting for the study of other superconducting sensors/detectors based on hot-electron effect, for example, hot-electron mixers.

## Methods

### Device fabrication

Ultrathin NbN films were synthesized through reactive DC magnetron sputtering of an 8-inch Nb target in an Ar + N_2_ gas mixture at room temperature. The typical background pressure was 9 × 10^−6^ Pa. The flow rates of Ar and N_2_ were set to 30 sccm and 4 sccm, respectively, under a total pressure of 0.27 Pa. The DC magnetron power was provided by current-regulated power supply set at 2.2 A with a resulting voltage of 270 V. The deposition rate was 6 Å/s, calibrated by X-ray reflectivity. The substrates used for film growth were Si (100) with its native oxide, MgO (100), MgF_2_ (110), Al_2_O_3_ (0001), and Si (100) with a 253-nm-thick buffering SiO_2_ layer. Prior to film deposition, all the substrates were cleaned by Argon ion-beam etching in a cleaning chamber. It is worth noting that the etching process removed native oxides of the Si (100) substrate. Because the MgO (100) and MgF_2_ (110) substrates allow the epitaxial growth of NbN films, NbN films on these substrates have higher critical temperature *T*_*c*_ than those on the Al_2_O_3_ (0001), SiO_2_/Si (100), and Si (100) substrates. To optimize the device performance, films with different thickness (*d*) values, i.e., 5.5, 4.5, 7, 7, and 7.5 nm were synthesized on the MgO (100), MgF_2_ (110), Al_2_O_3_ (0001), SiO_2_/Si (100), and Si (100) substrates, respectively. The films were structured into a conventional meandered nanowire structure, which covered a 15 μm-diameter area. All nanowire structures were patterned by electron-beam-lithography (EBL) with PMMA950-A2 as the e-beam resist. Electrode structures were formed by reactive ion etching (RIE) in the CF_4_ gas. Seven SNSPDs with different nanowire linewidth/space ratio (*w*/*s*) were fabricated. The detailed parameters of these SNSPDs are listed in Table 1.

### Measurement setup

**Figure 6:**
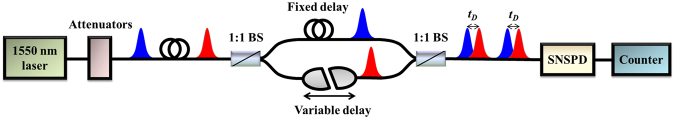
Schematic of the experimental setup for the *τ*_*th*_ measurements. Two fiber beam splitters (BS) and a motorized delay line were used to control the *t*_*D*_ between the two pulses.

The experimental setup for *τ*_*th*_ measurement is shown in Fig. 6. The source of optical excitation is a 20-MHz mode-locked fiber laser with a 1550-nm wavelength, 500-fs pulse duration, and a typical maximum average output power of 2 mW. Laser pulses are attenuated and then divided by a 1:1 fiber beam splitter. One part of the pulse is sent to a fixed fiber, whereas the other part is sent to a motorized delay line. The time delay *t*_*D*_ between the two pulses can be controlled by tuning the motorized delay line with a precision of ±0.01 ps. The two pulses are combined again on a 1:1 fiber beam splitter and then coupled to the SNSPDs. The measurements for the SNSPDs are performed in a Gifford–McMahon cryocooler at 2.15–5 K. A resistive heater is placed near the sample to tune the temperature of SNSPDs.
